# New Insights into Involvement of Low Molecular Weight Proteins in Complex Defense Mechanisms in Higher Plants

**DOI:** 10.3390/ijms25158531

**Published:** 2024-08-05

**Authors:** Magdalena Ruszczyńska, Hubert Sytykiewicz

**Affiliations:** Faculty of Natural Sciences, Institute of Biological Sciences, University of Siedlce, 14 Prusa St., 08-110 Siedlce, Poland; mr40@stud.uws.edu.pl

**Keywords:** plant defense, abiotic stress, biotic stress, dehydrins, cyclotides, heat shock proteins, pathogenesis-related proteins, thionins, defensins

## Abstract

Dynamic climate changes pose a significant challenge for plants to cope with numerous abiotic and biotic stressors of increasing intensity. Plants have evolved a variety of biochemical and molecular defense mechanisms involved in overcoming stressful conditions. Under environmental stress, plants generate elevated amounts of reactive oxygen species (ROS) and, subsequently, modulate the activity of the antioxidative enzymes. In addition, an increase in the biosynthesis of important plant compounds such as anthocyanins, lignin, isoflavonoids, as well as a wide range of low molecular weight stress-related proteins (e.g., dehydrins, cyclotides, heat shock proteins and pathogenesis-related proteins), was evidenced. The induced expression of these proteins improves the survival rate of plants under unfavorable environmental stimuli and enhances their adaptation to sequentially interacting stressors. Importantly, the plant defense proteins may also have potential for use in medical applications and agriculture (e.g., biopesticides). Therefore, it is important to gain a more thorough understanding of the complex biological functions of the plant defense proteins. It will help to devise new cultivation strategies, including the development of genotypes characterized by better adaptations to adverse environmental conditions. The review presents the latest research findings on selected plant defense proteins.

## 1. Introduction

**Figure 1:**
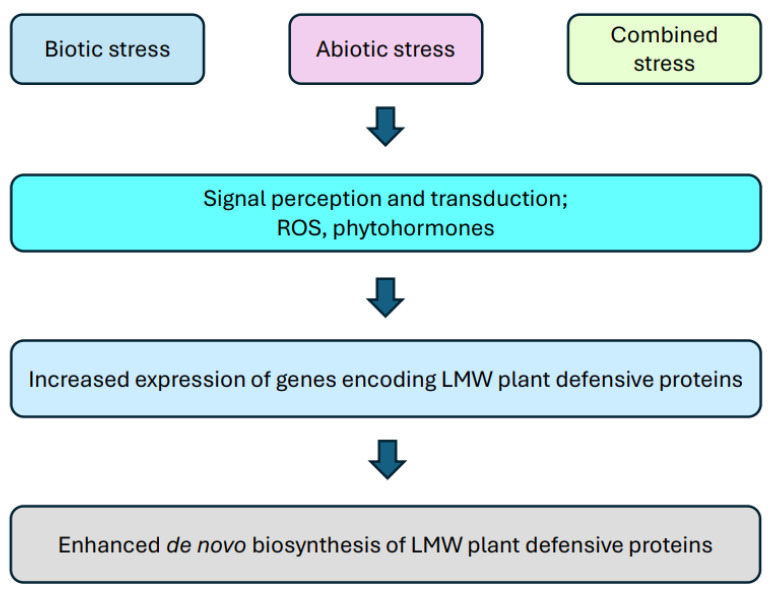
Scheme of stress-related induction of biosynthesis of the low molecular weight (LMW) defensive proteins in plants. ROS—reactive oxygen species.

Crop production is under threat because global warming is causing the climate to change dramatically. Plants experience a range of different stresses on a daily basis, both abiotic (e.g., drought, high/low temperature, salinity, flood, excess toxic metals) and biotic (e.g., pathogens, parasites, insects) [1]. Each of these stress factors results in reduced growth, yield and quality of the crop [2]. As a result, approximately 30% of the world’s major food crops are lost annually [3]. Unfortunately, this problem will grow as it is estimated that the intensity and frequency, as well as duration of environmental stressors, will increase [4]. This could negatively impact biodiversity and contribute to the threat of food security in an ever-growing global population [5]. Drought and salt stress are the most common abiotic stresses affecting plants. They severely restrict plant growth by drastically reducing the water content of plant cells and declining nutrient uptake [6]. Salt stress is also associated with a serious risk of salt ion accumulation (ion toxicity) [7]. In addition, after drought stress when re-watering occurs, secondary stresses, such as oxidative and osmotic stress, can be initiated [8]. During cold stress, changes occur in the cell membrane (the fluid within it turns into a semi-fluid gel or crystals). Moreover, protein folding can occur [9]. Although thermophilic plants exist, this does not mean that they are immune to heat stress. It is defined as an increase in temperature above the optimum temperature range for a certain period of time [10]. The faster the rate of temperature rise and the longer the duration of the stressor, the worse the effect on plant growth and development [11]. The plants most sensitive to heat are those at the reproductive stage, especially their male reproductive structures. In plants, many detrimental changes occur as a result of high temperatures, which may consequently lead to infertility [12]. In addition, high-temperature damages membrane proteins, leads to enzyme denaturation, accumulation of reactive oxygen species (ROS) and inhibits the photosynthesis process [13]. Constant access to sunlight exposes plants to ultraviolet B (UVB) radiation. UVB damages plant DNA, which interferes with photosynthesis and contributes to an increased production of phenolic compounds [14]. All these changes, which occur under the influence of both biotic and abiotic stressors, can lead to a lot of damage and eventually even death of the plant cell [13]. Plants, due to their sedentary lifestyles, have developed many mechanisms to amplify molecular signals to cope with and adapt to stressful conditions [15,16]. Short-term mechanisms aim to minimize sudden negative changes that occur in the plant cell during a stressful condition, while long-term mechanisms prepare the plant for stress experiences in the future [17]. Already at the first moment when a stressor starts to affect the plant, intensive changes take place. These may include strengthening the cell wall through lignification, suberisation or callose deposition. In addition to the accumulation of osmolytes, hormones activate the biosynthesis of proteins relevant to the stress response, such as late-embryogenesis abundant proteins (e.g., dehydrins), cyclotides, heat shock proteins and pathogenesis-related proteins (e.g., thionins and defensins), and this contributes to activating the plant’s defense mechanisms (Figure 1) [13,17,18,19,20,21,22]. These are complex processes, dependent on many factors, but a thorough understanding of plant defense mechanisms will contribute significantly to improving the quality and quantity of yields, despite unfavorable environmental conditions.

Importantly, it has been signally reported that specific low molecular weight defensive proteins may be involved in shaping cross-stress tolerance in plants exposed to heat, cold or osmotic stresses [23,24,25,26].

Protein posttranslational modifications (PTMs) play an extremely significant role in the regulation of the duration and intensity of almost all physiological processes in plants. It allows cells to respond dynamically to a variety of factors (exogenic and endogenic) [27]. PTMs can rapidly and yet reversibly affect existing proteins, altering their function, stability and location. The best-known chemical modification of proteins is phosphorylation, but it is also possible to modify proteins by conjugating target proteins to substrates via ubiquitination and SUMOylation. Ubiquitination and SUMOylation are associated with the attachment of ubiquitin and SUMO (Small Ubiquitin-like Modifier), respectively, to a lysine residue within the target protein [28]. SUMO participates in the regulation of plant growth and development and is involved in shaping tolerance to various environmental stresses (abiotic and biotic) [29,30,31]. It is a low molecular weight protein of about 100–115 amino acids (approx. 11 kDa) and is similar in structure to ubiquitin [32]. Ubiquitin is a low molecular weight protein of about 76 amino acids in length (approx. 8.5 kDa), which, with the proteosome, forms the ubiquitin-proteasome system (UPS) that plays extremely important roles in plant growth and development, including responses to environmental stressors [33]. SUMO and ubiquitin are processed prior to conjugation with the target proteins, which results in mature proteins having a similar tertiary structure [34]. For the most part, protein ubiquitination refers to the degradation of proteins through a mechanism of targeting modified (misfolded, aggregated) proteins to the 26S proteasome complex, where hydrolysis occurs with the release of ubiquitin [35]. In contrast, SUMOylation involves the formation of subnuclear structures, regulation of transcriptional activity, protein stability and DNA binding of transcription factors, as well as protein interactions with other proteins and DNA molecules [34].

The purpose of this review is to comprehensively discuss and interpret the latest and most advanced developments in uncovering the multifaceted and complex biological role of low molecular weight proteins in higher plants exposed to a broad range of stress factors.

## 2. Dehydrins

Dehydrins (DHNs) are one of the most important proteins involved in the plant defense response [36]. They belong to the class II proteins of the late-embryogenesis abundant protein (LEA) family. They are characterized by high hydrophilicity and thermostability [37]. These are low molecular weight proteins (9–200 kDa) [38]. Dehydrins contain many polar amino acid residues. They do not contain cysteine or tryptophan, but their polypeptide chain contains alanine or glycine [18]. The structure of the dehydrins (helix with a secondary structure) is only defined when a ligand is attached, which can be a membrane lipid or a metal ion [39]. Dehydrins contain the φ segment, the F segment, the N-terminal Y segment, the S motif and, the most essential, lysine-rich K segment (defines all dehydrins), without which the integration of dehydrins into other proteins would be impaired and protein aggregation could occur [18,40,41,42]. Under various stressors, DHN expression and accumulation are altered in both generative and vegetative organs [43]. 

To date, studies have shown a major defensive role of dehydrins during cold stress, acclimatization and deacclimatization (Table 1 and Table 2). In cold-tolerant varieties of both barley and wheat, a higher amount of dehydrins was observed in a shorter period of time, compared to varieties susceptible to this stress [44]. In two garden rose cultivars (i.e., Dagmar Hastrup and Chandos Beauty) characterized by different sensitivity to low temperature, a comparable increase in RhDHN5 production was stated with the length of exposure to this stress and a decrease in RhDHN5 synthesis during the deacclimatization process [45]. In the thale cress (*Arabidopsis thaliana* L.), the combination of the K segment of the cold-induced dehydrin Lti30 with lipid head groups through electrostatic interactions was shown to improve the cold tolerance of this plant [46]. The formation of membrane-protective aggregates was made possible by restricting the mobility of lipid molecules and associated proteins [47]. 

During drought stress, there is an increased accumulation of dehydrin proteins. This was confirmed by biotests conducted on white spruce (*Picea glauca* (Moench) Voss), in which a several-fold increase in the transcript levels of the dehydrins PgDHN10, PgDHN16, PgDHN33 and PgDHN35 was observed after several days of water shortage [48]. In addition, a statistically significant increase in dehydrins was observed when two wheat varieties with different resistance to this stressor were tested. In the resistant variety (Omskaya 35), there was a significantly higher accumulation of dehydrins, especially those of low molecular weight, compared to the drought-sensitive variety (Salavat Yulaev) [49]. Somewhat surprisingly, however, was a study on drought stress in rice (*Oryza sativa* L.). Expression of the DHN1 protein was downregulated by a decrease in dehydrin content in the plant subjected to this stress [50]. 

During the salt stress response of three halophytic species (i.e., *Puccinillia tenuiflora*, *Eutrema salsugineum* and *Hordeum marinum*), it was uncovered that there were differential plant responses to this stressor, depending on the structure of specific subclasses of dehydrins. It allowed for a better understanding of the mechanism by which these plants tolerate high concentrations of salt in the soil. This work revealed that many dehydrins are upregulated during salt stress and the intensity of this process is closely related to their structure. The FSKn and YnSKn subclasses of DHNs were most prevalent, and their expression levels were the highest of all tested, which may indicate that they are most closely associated with salt stress tolerance in these plants [51]. It was also revealed that soaking white clover (*Trifolium repens* L.) seeds in γ-aminobutyric acid (GABA) caused a higher production and accumulation of dehydrins, as well as an increase in transcriptional activity of the genes encoding SK2, Y2K, Y2SK and dehydrin b [52]. Moreover, in a study on rice (*Oryza sativa* L. ssp. *Indica*), which is the most salt-sensitive cereal, there was a significantly increased expression of dehydrins in the shoots after the stressor exposure [53]. The expression of the dehydrin gene *CdDHN4* under high and low temperatures, drought, salinity and abscisic acid (ABA) applications was studied in two cultivars of Bermuda grass (*Cynodon dactylon* L.): drought-tolerant (Tifway) and drought-sensitive (C299). In all these cases, there was an increase in *CdDHN4* expression, with ABA sensitivity of this gene, and its expression during drought stress was significantly higher in the tolerant variety [54]. In a survey conducted on the transgenic tobacco with four dehydrin genes (*PmLEA10*, *PmLEA19*, *PmLEA20* and *PmLEA29*) isolated from Chinese plum (*Prunus mume* Siebold and Zucc.), drought or cold stress was better tolerated by these plants, compared to the control (non-transgenic) plants. Transgenic plants subjected to both drought and cold stress showed significantly lower levels of malondialdehyde (MDA) and electrolyte leakage [55]. 

In order to reveal the role of the *CaDHN5* gene during abiotic stress in plants, the transgenic paprika (*Capsicum annuum* L.) plants with reduced expression of the gene under study were created; this was achieved by virus-induced gene silencing (VIGS) and transgenic *A. thaliana* plants that overexpressed *CaDHN5*. Plants with overexpression of the gene tested exhibited a greater tolerance to salt and osmotic stress, compared to wild-type plants. In contrast, higher ROS accumulation was observed in plants with reduced expression of the gene [56]. Similar studies were carried out for the *CaDHN3* gene (down- and overexpressed in *A. thaliana*) under salt stress and drought conditions. It has been reported that overexpression of the *CaDHN3* gene regulates osmotic stress responses in the transgenic plants by triggering the antioxidant mechanisms protecting plants from the detrimental effects of high amounts of ROS [57]. Equally promising effects of dehydrins were investigated during combined salt and cold stress. The study used the dehydrin gene *CaDHN4*, isolated from *C. annuum* leaves and overexpressed in the *A. thaliana*. Plants with *CaDHN4* overexpression had much less lipid peroxidation (lower MDA content) and electrolyte leakage, compared to the wild type [58]. Transgenic tobacco overexpressing the dehydrin gene *SbDHN1* from sorghum (*Sorghum bicolor* (L.) Moench) was able to survive under high temperature and osmotic stress (conditions that occur during drought stress) for 15 days. It is the first report confirming the protective effect of this gene on the plant proteome under the applied stress conditions [59]. Biotests carried out on the transgenic tobacco with overexpression of the *SiDHN* dehydrin gene isolated from the snow lotus (*Saussurea involucrata* (Kar. and Kir.)) demonstrated that the gene enhances tolerance of the transgenic plants to drought and cold stresses. Similarly, overexpression of the *SiDHN* dehydrin gene improved the tolerance of the transgenic tomato (*Solanum lycopersicum* L.) to these two abiotic stresses. It was achieved by increased scavenging of ROS by enhanced biosynthesis and activity of stress-related antioxidative enzymes that declined the cell membrane damage and improved the chloroplasts’ integrity [60]. 

Studies suggest that dehydrins, as plant defense proteins, play a particularly significant role during stress conditions. They positively regulate signaling pathways during low temperature, drought, salt and osmotic stresses. It creates an important opportunity for genetic engineering to develop new plant genotypes displaying a higher degree of resistance/tolerance to the above-mentioned stressors. 

## 3. Cyclotides

Cyclotides are macrocyclic peptides synthesized from precursor proteins on ribosomes [92]. Cyclotides have a cyclic head-to-tail peptide backbone of approximately 30 amino acids [61]. In addition, they have three disulphide bridges in a nodal conformation (one disulphide bridge crosses the macrocycle comprising the other two disulphides and links the peptide backbones together), named the cyclic cysteine knot (CCK) [48,93]. This makes cyclotides extremely stable and resistant to biological, chemical and physical factors (e.g., high temperatures, extreme pH or enzymatic degradation) [94]. However, up to date, there are no studies assessing their stability directly in plant cells [95]. These proteins are unique in plants, although cyclic peptides are also present in other organisms [96]. They are synthetized by plants within at least six angiosperm families, including Cucurbitaceae, Fabaceae, Poaceae, Rubiaceae, Solanaceae, and Violaceae [97,98,99]. It is possible to produce them in transgenic plants (e.g., tobacco, oilseed rape, lettuce, bush beans) by co-expressing the target peptides with aspartic endopeptidase cyclases (AEPs) [100]. 

Unfortunately, at this point, little is yet known about the exact role they play in plants in which they are synthesized. It is likely that cyclotides are produced and accumulated in all organs and tissues of a given plant in response to the prevailing environmental conditions. They are quite abundant, and their distribution may be related to their functions [101]. It is estimated that the plant is able to synthesize and accumulate up to 1.5 g of cyclotides per 1 kg of fresh weight, which is certainly a very demanding process for the plant. At the same time, this may indicate that cyclotides are very valuable biologically, and their presence in organs, tissues and cells that are most vulnerable to attack by pests and pathogens suggests that they may act as defense proteins [19,62]. Interestingly, it appears that each plant species may be characterized by a different set of cyclotides, making it possible to distinguish these species from each other based on their distribution and content [97]. In the vast majority of studies, cyclotides were extracted from the plants and exposed to the examined stress factors [61].

A study on the wood violet (*Viola odorata* L.) showed that mite foraging activates cyclodiene production, with significantly higher levels than in the control plants [102]. In the suspension culture of the swamp violet (*Viola uliginosa* Besser), the effect of stress hormones and biological elicitors on cyclotide synthesis was investigated. The biotests showed a statistically significant increase in the production of three cyclotides (viul M, cyO13 and cyO3) in suspensions with different concentrations of jasmonic acid (50, 100 and 200 µM) after 14 days of culture [61]. Extensive studies were carried out on the response of maize (*Zea mays* L.) to selected abiotic stressors (mechanical injury, drought and salinity), biotic stressors (*Gibberella zeae* (Schwein.) Petch, *Ustilago maydis* (DC.) Bref. and *Rhopalosiphum maydis* (F.)) and elicitors (i.e., salicylic acid and methyl jasmonate). The survey was focused on the *Zmcyc1* and *Zmcyc5* genes whose expression was confirmed in all tissues examined (the highest in leaves and the lowest in roots). Mechanical injury (by cutting the leaf blade with a razor blade) and the use of elicitors significantly increased the expression of both genes tested, while higher expression was observed for *Zmcyc1*. Biotic stressors and salt irrigation also affected the increased expression levels of the genes evaluated, with no significant differences between *Zmcyc1* and *Zmcyc5*. During drought, there was an increase in the expression of both genes assessed, while higher expression was observed for the *Zmcyc5* gene (Table 1) [63]. 

**Figure 2:**
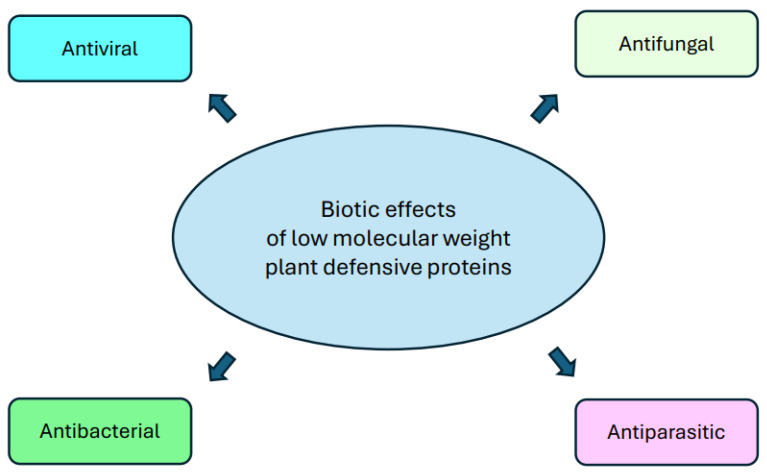
Biotic activity of the low molecular weight plant defensive proteins.

Cyclotides have been extensively studied for their biological activity, which could be potentially useful in bioengineering, for the development of new drugs. So far, they are the only ones among plant peptides to have bioavailability after oral administration [103]. They have been shown to be capable of disrupting phospholipid membranes by selectively binding to specific lipids [104]. As a result, they exhibit antimicrobial (especially in the case of Gram-negative bacteria), antifungal, antiviral, hemolytic, cytotoxic effects and may be effective against cancer cells (Figure 2) [105,106]. In addition, their use as a scaffold in drug design in the treatment of obesity, Alzheimer’s disease and autoimmune diseases, including the central nervous system demyelinating disease multiple sclerosis (MS), is proving extremely promising [107,108,109,110]. Cyclotides have a number of potential applications in the treatment of various human and animal diseases. Furthermore, few studies demonstrated their effective use as biological plant protection products (biopesticides) [111,112]. 

Cyclotides are therefore a promising group of defense plant proteins with multifaceted applications. Although their role in the host organism is not fully known, they could become a powerful tool to improve the health of humans and animals, as well as to boost plant production. 

## 4. Heat Shock Proteins 

Heat shock proteins (HSPs) are known as molecular guardian proteins. Depending on their molecular weight (in the range approx. 8–200 kDa), amino acid sequence homology, activity and function, they can be divided into six types: sHSPs (small heat stress proteins), HSP40, HSP60, HSP70, HSP90 and HSP100 [67,113]. Plants have thermosensors that enable them to recognize specific changes and activate appropriate defense mechanisms in response (Table 1 and Table 2) [64]. When a plant is subjected to heat stress (HS) involving a sudden change in temperature, thermosensors are activated (change in membrane fluidity, fragmentation of nucleic acids, denaturation of proteins), and it is sensed by heat shock factors (HSFs), and next, the expression of HSPs is activated [13,114]. HSP expression may also be upregulated during plant tissue damage, pathogen attack, water deficiency/excess, radiation and low-temperature stress [65,66]. 

Heat shock proteins are found in the cytoplasm, cell nucleus, mitochondria, chloroplasts and endoplasmic reticulum [67]. The functions of HSPs are associated with the cell cycle control, formation of multiprotein complexes, transport, translocation, folding (of newly formed or damaged proteins), unfolding and degradation of proteins [115]. The quantity and quality of HSPs synthesized depends on several factors, including the type, developmental stage and degree of differentiation of the plant cell or tissue, as well as the temperature altitude and duration of the stressor [67]. A feature of the acquisition of heat tolerance is the mass production of HSP [116]. Changes in the concentrations of heat shock proteins may serve as biomarkers of oxidative stress associated with the presence of heavy metals. It has been proved that exposure of plants to cadmium ions may cause a several-fold increase in the expression of the *HSP70* and *HSP27* genes [68]. In the study conducted on radish (*Raphanus sativus* L.), 34 *RsHSP70* genes were identified. Characterizing their expression patterns allowed the authors to understand their roles in the growth, development and plant responses to abiotic stress (high/low temperature, cadmium exposure, drought, high salinity) and biotic stress (*Plasmodiophora brassicae* infection). Under stress conditions, there was an increased expression of the *RsHSP70-23* gene in the plants, indicating its significant involvement in the defense responses [69]. 

The heat shock protein gene *TaHSP17.4* was identified in wheat, and its expression was induced by heat, drought and salt stress. Under stress conditions, plants with overexpression of this gene showed lower MDA content and higher proline amount. The plants tested had a much higher tolerance to heat, salinity and drought stress than the wild-type plants [70]. Furthermore, five durum wheat cultivars (*Triticum durum* Desf.; J2, A8, T11, M23 and R15) were subjected to salinity stress and the expression of the four HSP genes (*Hsp17.8*, *Hsp26.3*, *Hsp70* and *Hsp101*), selected stress parameters (i.e., proline, chlorophyll, and MDA content) and plant growth were examined. A reduction in plant growth was noted in all the cultivars. Genotypes T11 and M23 were at the highest degree of adaptation to the tested stress, as they had high expression levels of HSP proteins, the lowest level of stress parameters and their growth was least inhibited. Conversely, the cultivars J2 and A8 performed much worse under salt stress conditions—HSP gene expressions were low, they had the highest level of the quantified stress parameters and their growth was drastically suppressed [71]. The response of small heat shock proteins (LsHsps) in lettuce (*Lactuca sativa* L.) to UV radiation and high-intensity light stress was investigated. The study showed a strong response at the transcriptional level in the *LsHsps*, *LsHsp60s* and *LsHsp70s* genes. In contrast, the light stimulus did not elicit any response in the *LsHsp90s* and *LsHsp100s* genes [72]. The overexpression of the *ZjHsp70* gene isolated from Japanese eelgrass (*Zostera japonica* Asch. and Graebn) and inserted into *A. thaliana* was examined. The transgenic plants exhibited enhanced heat tolerance due to increased activity of the antioxidant enzymes and lower MDA content than the wild variety [73]. The expression of the *CaHsp25.9* gene was investigated in two *C. annuum* lines, thermotolerant (R9) and thermosensitive (B6), under heat stress. In both lines, there was a strong induction of the gene under stress. In line R9, the expression of the tested gene was also induced by salt and drought stress. In contrast, silencing of this gene increased the accumulation of MDA and ROS in the test line. The same gene was overexpressed in *A. thaliana* plants, resulting in reduced MDA content, increased activity of antioxidative enzymes and upregulation of stress-related genes during drought, heat and salt stress [74]. 

A newly identified gene *AsHSP26.8a* in the creeping bentgrass (*Agrostis stolonifera* L.) was cloned and overexpressed in the transgenic *A. thaliana*. It turned out that the plants with overexpression of this gene exhibited a declined tolerance to both heat and salinity stress, in addition to hypersensitivity to the ABA hormone [75]. In contrast, in a later study, overexpression of the *AsHSP26.2* gene from the same plant had a beneficial effect on the growth and development of the transgenic plant [76]. The novel *HvHSP16.9* gene was investigated under salt stress. It was cloned from wild barley (*Hordeum spontaneum* (Koch) Thell) and inserted into the transgenic *A. thaliana*. Overexpression of this gene increased salt tolerance in the plants tested [77]. Moreover, overexpression of the *NtHSP70-8b* gene in tobacco (*Nicotiana tabacum* L.) elevated seed biomass, altered stomatal conductance and enhanced antioxidative systems in the leaves, thus improving heat stress tolerance [78]. It was also unveiled that overexpression of the small heat shock protein GmHSP18.5a during high-temperature stress increased fertility in a male cytoplasmic male-sterility (CMS)-based regenerator line in soybean. The mechanism of action was based on an enhancement of the antioxidative system and an efficient uptake of ROS [79]. 

The presence of HSPs has been confirmed in all living organisms. The plant-derived HSPs were assessed in the context of their therapeutic properties in neurodegenerative diseases in humans [117]. In addition, through knowledge of the mechanisms of HSP expression, it is possible to regulate the flowering process in plants [118,119]. In addition, the expression of heat stress proteins is being studied in plant pests in order to be able to use more environmentally friendly plant protection products in the future [120,121]. Moreover, based on knowledge of HSP expression, it is possible to select appropriate feeding methods for breeding animals in order to reduce the negative effects of the heat stress they experience [122,123].

Various HSP encoding genes have been extensively examined in transgenic plants subjected mostly to heat, drought and salinity stress. The plants showed greater tolerance to the applied stressors through activation of the antioxidative systems, thus declining ROS and MDA contents. 

## 5. Pathogenesis-Related Proteins

In plants, the production and accumulation of pathogenesis-related (PR) proteins occur in response to biotic stressors. So far, 19 PR protein families (PR-1 to PR-19) have been identified [80]. Representatives of these families are not characterized by sequence similarity but may be classified by common biochemical features. These include low molecular weight (6 to 43 kDa), antimicrobial activity, hydrophobic cavities and ligand-binding ability [124]. Of the 19 families, as many as eight cause allergic reactions in humans [125]. 

The mechanism of action of the antifungal proteins (PR-1) is based on the binding and sequestration of sterols from the pathogen, disrupting its further growth [80]. Hydrolytic β-1,3-glucanase (PR-2) and chitinases (PR-3, PR-4, PR-8, PR-11) destroy fungal cell walls by disassembling their main components [20,81]. Thaumatins (PR-5) inhibit the growth of fungal hyphae and spores by forming pores in cell membranes, thereby leading to electrolyte leakage, while endoproteinases (PR-7) decompose the fungal cell walls [80]. The mechanism of action of peroxidases (PR-9) is associated with the deposition of lignin in the plant cell wall, thereby strengthening it [82]. Ribonucleases (PR-10) activate plant cell apoptosis and hypersensitivity reactions (HRs) [126]. Oxalate oxidase proteins (PR-15 and PR-16) are essential during the generation of excessive ROS amounts and the oxidative burst [127]. The pine antimicrobial protein Sp-AMP (PR-19) is able to alter the structure of the fungal cell wall through glucan binding [83]. In addition to these, pathogenesis-related proteins also include antimicrobial peptides (AMPs), such as proteinase inhibitors (PR-6), which can inhibit the viral replication cycle and quinine synthesis in fungal cell walls, defensins (PR-12), thionins (PR-13) and lipid transfer proteins (PR-14), which exhibit antimicrobial and antifungal properties by altering the membrane permeability [80,128]. Moreover, secretory proteins (PR-17) and carbohydrate oxidases (PR-18) exhibiting antimicrobial activity were identified (Table 1 and Table 2) [80]. Furthermore, it has been reported that the osmotin belonging to the PR-5 protein family may act as a potential therapeutic drug for humans, being an agonist of adiponectin [84].

Pathogenesis-related proteins occur in all plant organs. For example, in leaves, they may account for up to about 10% of the total protein [129]. They are crucial in the initial stage of stress associated with pathogen infection, being the first line of defense against these stressors. As a result, it is possible to minimize the damage caused by pathogens [20]. These proteins are thermostable and protease-resistant [130]. PR proteins are expressed under a stress stimulus, such as a pathogen attack, exposure to elicitors and contact with excessively high concentrations of plant hormones [124]. They are responsible for systemic acquired immunity (SAR) and the hypersensitivity response (HR) to counterattack by pathogens (fungi, bacteria, and viruses). SAR is activated by contact with a pathogen that triggers a cascade of signals, inducing the synthesis of PR proteins. This type of resistance is highly desirable because it can be active in the plant for up to several days; moreover, it is possible to transmit this resistance to the next generation of plants [131,132]. The hypersensitivity reaction (HR) involves the rapid death of the directly affected cells and those adjacent to them so that the spread of the pathogen becomes impossible (the pathogen loses its food source) [133]. 

The best-characterized PR proteins are thionins and defensins, which are described below.

### 5.1. Thionins 

Thionins are a family of plant proteins belonging to AMPs (antimicrobial peptides). They are small peptides with a molecular weight of about 5 kDa and sizes ranging from 45 to 48 amino acid residues [134]. Thionins are synthesized as precursor proteins, which include a signal peptide, a mature basic domain and a C-terminal acidic prodomain [135]. They are found in both mono- and dicotyledonous plants [136]. Thionyls are divided into five classes depending on the charge and number of cysteine residues (eight residues—class I and II; six residues—class III, IV and V). The sequences and secondary structures of thionins are highly conserved. As for the spatial structure of thionins, they have the shape of the large Greek letter gamma “Γ” (vertical arm—two antiparallel α-helices; horizontal arm—an elongated coil and a short antiparallel β-sheet) [135]. Thionins are considered plant toxins because they show toxic effects against bacteria, fungi, yeasts and insects [137]. Overexpression of thionins has been shown to increase plant resistance to various pathogens [138]. The likely mechanism of action of thionins is to open pores in the cell membranes of the pathogens, leading to leakage of calcium and potassium ions from their cells [139]. 

Nine proteins of the thionin family were isolated from the seeds of black caraway (*Nigella sativa* L.) and named ‘nigellothionins’. Three of them were tested for antifungal properties. During in vitro studies, one of them, designated NsW2, showed high cytotoxicity already at submicromolar concentrations, and this means that it may have applications in antifungal and antiproliferative agents [140]. A study was performed in which modified thionin (mthionin) was cloned in transgenic *A. thaliana*. The plants were infected with *Fusarium graminearum* (a fungal pathogen that causes ear blight). The transgenic plants expressing mthionin showed suppressed fungal development (lower fungal biomass in leaves and inflorescences) compared to the control plants [85]. Moreover, the expression of 44 *O. sativa* thionins labeled as OsTHION was checked in silico. By modulating their expression levels, their active involvement in responses to biotic and abiotic stresses was established. One of them, *OsTHION15*, was recombinantly expressed in *Escherichia coli* and further tested to verify its antimicrobial activity. The results confirmed its inhibitory activity against bacteria and viruses pathogenic to rice. In addition, studies of the expression of this thionin in transgenic tobacco (*Nicotiana benthamiana* Domin) were carried out, which also confirmed its potential use in plant disease control [90]. Moreover, two barley genotypes (i.e., aphid-susceptible Concerto cv. and partially resistant to the aphids Hsp5 cv.) were tested for drought and the bird cherry-oat aphid (*Rhopalosiphum padi* L.) feeding. During drought, an increased expression of the thionin gene *HvTHIO1* occurred in Hsp5 plants that negatively affected the aphid’s development [86]. 

An experiment was conducted to investigate responses of *A. thaliana* plants to individual and combined abiotic stresses (heat and osmotic) followed by infection with biotrophic (*Pseudomonas syringae* pv. *tomato*) and necrophytic (*Botrytis cinerea* Pers.) pathogens. The data collected showed that when a plant first experienced abiotic stress, there was a reduced expression of the defense protein genes, including thionin 2.2 (*thi2.2*), and thus, later plants were more susceptible and less able to cope with biotic stressors [141]. Two thionin genes (*AT1G12660* and *AT1G12663*) from *A. thaliana* were transformed into two potato cultivars (i.e., Lady and Spunta). The transgenic potato varieties showed much greater resistance to the fungal infection (*Fusarium solani* and *Fusarium oxysporum*), and a lower number of germinating fungal spores was observed compared to the control [142]. Transgenic citrus plants (Carrizo—hybrid of Washington Navel orange and *Poncirus trifoliata*) overexpressing a modified thionin gene were treated with the bacterial pathogen *Xanthomonas citri* that causes citrus cankers. The results showed a reduction in the disease symptoms and inhibition of bacterial growth in relation to the control. Equally promising results were obtained against the bacterium *Candidatus Liberibacter asiaticus*, which causes the serious disease—Huanglongbing (HLB) citrus greening [143]. Furthermore, an increased expression of the rice thionin *OsThi9* (similar to defensin) under cadmium exposure was demonstrated. Overexpression of this thionin resulted in a higher accumulation of cadmium in the cell wall, thus preventing the movement of this metal into the shoots. In plants growing on soils contaminated with this heavy metal, it reduced cadmium accumulation and this was performed without harming the plants [87]. 

Thionins are a significant group of plant defense proteins that could be successfully used in plant protection against viral and bacterial diseases. In addition, they appear promising for mitigating heavy metal toxicity.

### 5.2. Defensins 

Defensins represent one of the largest families of plant antimicrobial peptides with a broad spectrum of bioactivity [144]. They are small cationic proteins rich in cysteine (Cys) and the basic amino acids lysine (Lys) and arginine (Arg). Defensins are characterized by a highly conserved and stable structure; a CSαβ conformation is present in them formed by four pairs of cysteines forming disulphide bonds that stabilize the α-helix and three β-fold sheets [144]. Despite the conserved conformation of CSαβ, the amino acid sequences of the primary structure may vary considerably [145]. They are stable at extreme values of pH and temperature [146]. Less common but with high biological activity are also histidine-rich defensins (HRDs) [147]. 

The vast majority of plant defensins are components of the innate immune system. They are mostly found in seeds, while their presence has also been confirmed in leaves, flowers and fruits [21]. Defensins have a wide range of biological functions. However, they are best known for their antimicrobial activity against bacteria (Gram-positive and Gram-negative), fungi, viruses and parasites [148]. But their activity is not limited to a response to plant pathogens, and their mechanism of action ranges from the interaction with specific lipids to the generation of ROS to induction of programmed cell death [149]. 

Atypical expression of plant defensins is associated with increased tolerance to biotic and abiotic stresses. A study conducted on *A. thaliana* evidenced that some of the seven members of the plant defensin 1 (AtPDF1) family could increase plant tolerance to excess zinc (Zn) accumulation. In addition, these proteins enhance the plant responses to necrotrophic fungi [88]. In a study of defensin expression under abiotic stress (mechanical injury), biotic stress (*Macrophomina pseudophaseolina* fungal infection) and the combined effects of these stresses in cassava (*Manihot esculenta* Crantz), five candidates of defensin genes (*MetDef*) in the genomic sequence were identified. Only *MetDef1* and *MetDef2* genes occupied adjacent positions on the same chromosome arm. It was noted that the expression of *MeDef1* and *MeDef5* genes was induced in leaves in response to a single abiotic and biotic stress but combined stresses (injury+fungus) did not have this effect. During all stress combinations, only the expression of *MeDef3* in root tissues was upregulated. In contrast, there was a downregulation of *MeDef2* in stem tissue during all stress combinations [89]. The chickpea (*Cicer arietinum* L.) *Ca-AFP* defensin gene was cloned and transformed into *A. thaliana*. Next, the plants were exposed to mannitol and polyethylene glycol-6000 in order to induce water deficit conditions. The results demonstrated that overexpression of the *Ca-AFP* gene in leaves of the transgenic *A. thaliana* enhanced tolerance to the water deficit stress. The applied stress conditions reduced the transpiration rate and stomatal conductance. In contrast, they increased water use efficiency and intensity of the photosynthesis process. Compared to the control, the transgenic plants had a lower electrolyte leakage and MDA content and, conversely, a higher content of proline, relative water, chlorophylls and increased activity of oxidoreductases, such as catalase (CAT), superoxide dismutase (SOD) and ascorbate peroxidase (APX) [91]. The plant defensin *AtPDF2.6* has been shown to be localized in the cytoplasm and it was not secreted into the apoplast. Its expression occurs mainly in root xylem parenchyma cells when exposed to cadmium (Cd). Overexpression of *AtPDF2.6* increases Cd tolerance in *A. thaliana* by stimulation the chelation reaction [150]. 

The defensin 8 gene (*DEF8*) is strongly expressed in *O. sativa* grains. Studies have shown that this gene is involved in the long-distance transport of cadmium (Cd) from the root to the shoot, and its chelating properties favor the removal of excess of this heavy metal from the rice grains. The mode of action of this gene in the transgenic *A. thaliana* confirmed that it may be used to control Cd accumulation also in other plant species [151]. A study of the defensin gene *AtPDF1.5* under excess Cd and low nitrogen stress conditions in *A. thaliana* revealed that the gene is the essential component of signal transduction, regulation of Cd removal and adaptation to low nitrogen levels [152]. 

Defensins are produced by all plant species, vertebrates and invertebrates [153]. Uncovering the biosynthetic routes of these proteins in plant pests may help to control their populations more effectively [154]. Plant defensins play a pivotal role during heavy metal (e.g., cadmium, zinc) stress, drought and pathogen infection. By overexpressing the genes encoding these proteins, it is possible to increase the efficiency of plant adaptation and survival under stressful conditions.

## 6. Conclusions

Deciphering the expression mechanisms of low molecular weight plant defensive proteins related to abiotic and biotic stresses will allow new cultivation strategies to be planned, including the developing of genotypes that may cope with the detrimental conditions associated with climate changes. The newly created plant varieties may provide a greater source of food, as the plants will have augmented tolerance to a variety of environmental stressors. Furthermore, it will be possible to synthesize new drugs of medical potential, as well as plant protection products, that are less toxic but equally or more effective, compared to current preparations. Further studies should also include protein-protein interactions in order to uncover the complexity of biological functions of low molecular weight defense proteins in plant cells. 

## Figures and Tables

**Table 1:** Summary of selected groups of low molecular weight proteins involved in defense responses of plants.

Groups of Proteins	Stress Factors Affecting Plants	References
Dehydrins	Drought; Low temperature; Osmotic stress; Salt stress.	[44,45,46,47,48,49,50,51,52,53,54,55,56,57,58,59,60]
Cyclotides	Jasmonic acid, methyl jasmonate and salicylic acid treatments; Pathogen attack; Pest infestation; Mechanical injury.	[61,62,63]
Heat shock proteins	Drought; Heavy metal stress; Heat stress; Low temperature; Salt stress; UV radiation; Mechanical injury; Pathogen attack.	[64,65,66,67,68,69,70,71,72,73,74,75,76,77,78,79]
Pathogenesis-related proteins	Heavy metal stress; Mechanical injury; Pathogen attack; Pest infestation.	[80,81,82,83,84,85,86,87,88,89]

**Table 2:** Examples of enhancement of abiotic and biotic stress tolerance of the transgenic plants overexpressing genes encoding the low molecular weight defensive proteins.

Cloned Gene	Source Plant Species	Target Plant Species	Increased Tolerance to the Specific Abiotic and Biotic Stresses	References
*CaDHN5* *(dehydrin)*	*Capsicum annuum* L.	*Arabidopsis thaliana* L.	Salt and osmotic stress	[56]
*CaDHN4* *(dehydrin)*	*Capsicum annuum* L.	*Arabidopsis thaliana* L.	Salt and cold stress	[58]
*CaDHN3* *(dehydrin)*	*Capsicum annuum* L.	*Arabidopsis thaliana* L.	Osmotic stress	[57]
*SbDHN1* *(dehydrin)*	*Sorghum bicolor* (L.) Moench	*Nicotiana tabacum* L.	High-temperature and osmotic stress	[59]
*SiDHN* *(dehydrin)*	*Saussurea involucrate* Kar. and Kir.	*Solanum lycopersicum* L.	Drought and cold stress	[60]
*ZjHsp70* *(heat shock protein)*	*Zostera japonica* Asch. and Graebn	*Arabidopsis thaliana* L.	High-temperature stress	[73]
*CaHsp25.9* *(heat shock protein)*	*Capsicum annuum* L.	*Arabidopsis thaliana* L.	High-temperature, salt, and drought stress	[74]
*HvHSP16.9* *(heat shock protein)*	*Hordeum spontaneum* (Koch) Thell	*Arabidopsis thaliana* L.	Salt stress	[77]
*OsTHION15* *(thionin)*	*Oryza sativa* L.	*Nicotiana benthamiana* Domin	Bacterial and fungal infection	[90]
*Ca-AFP* *(defensin)*	*Cicer arietinum* L.	*Arabidopsis thaliana* L.	Water-deficit stress	[91]

## Data Availability

Data sharing is not applicable (only appropriate if no new data is generated or the article describes entirely theoretical research).
